# Early Undifferentiated Arthritis: A Developing Country Perspective from Nepal

**DOI:** 10.31729/jnma.3893

**Published:** 2018-12-31

**Authors:** Binit Vaidya, Rikesh Baral, Shweta Nakarmi

**Affiliations:** 1 Department of Rheumatology, National Center for Rheumatic Diseases, Ratopul, Kathmandu, Nepal

**Keywords:** *arthritides*, *Nepal*, *power doppler*, *rheumatoid*, *ultrasonography*

## Abstract

Early undifferentiated arthritis is a group of inflammatory joint disease of less than 3 months duration that do not classify under any of the specific rheumatic or connective tissue disorder. Previously, inflammatory arthritis used to be treated only when there was a clear evidence of damage or deformity occurring with it. Use of disease modifying anti-rheumatic drugs were considered potentially harmful early in the course of arthritis which could be self-limiting. However, with the abundance of data on outcomes of early arthritis and identification of factors that can help to predict those outcomes lead to earlier use of such DMARDs. Better understanding of serological tests like anti-CCP antibodies and imaging modalities like high frequency ultrasound with power doppler and magnetic resonance imaging has increased the diagnostic and prognostic yield of such early arthritis cases. It is now imperative that the risk be assessed early in the course of disease and early DMARDs be instituted for better outcome in these cases.

This review analyses the historical evolution of evidence in the management of early undifferentiated arthritis and summarises the treatment approach, monitoring and disease outcomes till date.

## INTRODUCTION

Early inflammatory arthritis (EIA) is defined as an inflammatory joint disease of less than 3 months duration.^1^ EIA are usually undifferentiated arthritis (EUA),^2,3^ meaning that it does not fulfil the criteria for any defined inflammatory or connective tissue disease. However, EIA can be an early presentation of rheumatoid arthritis (RA) or other rheumatic diseases too.^2,3^ After the updated classification criteria from joint collaboration between EULAR/ACR in 2010,^4^ we are able to diagnose many cases of EIA as early rheumatoid arthritis. The management protocol is well defined once a patient with EIA is classified as rheumatoid arthritis. Management still remains confusing and challenging when we encounter cases of EIA who do have definite features of inflammatory joint disease early in the course and yet still do not classify as established rheumatoid arthritis.

This review intends to summarize the available evidence for the logical management of early undifferentiated arthritis.

## BACKGROUND

Early undifferentiated arthritis constitutes a group of inflammatory joint disease of less than three months duration which do not fulfil any of the classification criteria for established rheumatic diseases,^2^ especially rheumatoid arthritis. By definition, patients with EUA should at least have one swollen or tender joint.^4^ Those patients with only stiffness, arthralgias or painful movement without any swelling or tenderness cannot be classified as undifferentiated arthritis. Many cases of early rheumatoid arthritis, scleroderma or lupus might present as EUA and the diagnosis usually becomes apparent over the period of next few months or even a year.^5^ About one third of the patients with EUA are believed to progress to rheumatoid arthritis or any other established rheumatic disease;^6^ another one-third will remain as undifferentiated arthritis and the remaining third is expected to go into spontaneous remission.^7^ This proportion is likely to change with the adaptation of more sensitive markers for prediction of the persistence of arthritis. Application of new 2010 classification criteria for rheumatoid arthritis has led to an increase in the diagnosis rates thus decreasing the proportion of cases which remain undifferentiated.^8,9,10^ Also, more sensitive imaging tests like high-frequency ultrasonography with power-doppler uptake and extremity magnetic resonance imaging have shown to identify the cases of inflammatory arthritis early before it is clinically apparent.^11,12^

## HISTORY OF THE WINDOW OF OPPORTUNITY

After late 90s, the concept of therapeutic window of opportunity was explored by many authors. Emery, in his review article in 1995, mentioned that it is beneficial to treat patients with poor prognostic factors with aggressive treatment and it is still appropriate to treat patients who do not fulfil the then ACR 1987 criteria for rheumatoid arthritis with disease modifying drugs (DMARDs).^13^ Early initiation of treatment may also alter the course^14,15^ and radiographic progression of disease.^16^ Studies were then conducted to identify those patients who will have persistent disease and thus would require DMARDs in future. It was found that the disease duration at the beginning of treatment was the most important factor that determined the persistence.^17^ It was seen that patients who were treated early in the course of their disease had better outcomes compared to those who had a disease duration of more than 1 year.^18^ A review by Dixon and Symmons tried to define the life cycle of rheumatoid arthritis comprising of four phases: phase of onset, phase determining persistence or remission, phase of evolution to a definite form and phase of severity or complication.^2^ They postulated that proper intervention at the phase that determined persistence could have dramatic change in the outcome of early arthritis.

Methotrexate was shown to have beneficial effects with delayed diagnosis of established rheumatoid arthritis and retardation of radiographic damage when given in cases of undifferentiated arthritis.^19^ In 2007, the Leiden prediction rule was developed after studying the factors that predicted the persistence of arthritis in the cohort from Leiden early arthritis clinic comprising of around 1700 patients completing one year of follow up. This model took into account nine variables namely sex, age, localization of symptoms, morning stiffness, the tender joint count, the swollen joint count, the C-reactive protein level, rheumatoid factor positivity, and the presence of anti-cyclic citrullinated peptide antibodies with varying weights to predict the persistence of arthritis and thus helping to identify candidates for methotrexate treatment.^20^

The most convincing evidence for the presence of time-limited window of opportunity came from a study by Neis et al in ESPOIR and Leiden cohorts showing a non-linear relationship between disease persistence and disease duration that suggested the presence of a limited time period where RA is more susceptible to treatment. This period was identified as 19.1 and 14.9 weeks respectively in the two cohorts. The evidence thus gradually evolved from suggestion to definition of the early time limited therapeutic opportunity when the disease is more amenable to treatment and when a proper intervention can change the natural course of disease from persistence to remission. ^21^

## EARLY DIAGNOSIS OF INFLAMMATORY ARTHRITIS

As described earlier, the presence of early window of opportunity was being studied from very early years. But back then, rheumatoid arthritis was diagnosed based on persistence and chronicity and thus by definition would take a long time to diagnose. ^22^ This long time before confirming the need of DMARDs, which were then thought to be toxic and to be used only when patient showed features of chronicity, was difficult to decide. Histological studies of the synovial tissue failed to show any difference in the nature and immune phenotype of the cellular infiltrates between early and late RA.^23^ After the availability of the anti-CCP antibody testing in early 2000, few prediction rules and prognostication were tried as mentioned before. After the 2010 EULAR/ACR criteria, many patients who would remain undifferentiated with older criteria were classified as rheumatoid and treated aggressively.^24^ However, there still was a significant number of patients who remained as undifferentiated.

MRI of the extremities was considered a sensitive tool to detect subclinical synovitis.^25–27^ It was shown that the presence of bone marrow edema on MRI correlated will future development of erosive disease.^26^ The finding was more significant in a clinically matching patient with additional finding of effusion and osteitis. The test was recommended as help to clinical detection of synovitis in the 2007 EULAR guidelines^28^ on early arthritis but, because there were high false positive results with MRI due to artefacts confusing with marrow edema and also due to inhibitory cost and unavailability in many settings,^29^ it was removed from the 2016 update of the same recommendation.^3,29^

Musculoskeletal ultrasonography with power doppler scanning has replaced the MRI in the recent update of recommendations by EULAR.^3, 25, 30^ With an experienced operator and a high frequency probe, USG can detect synovial hypertrophy, effusion, soft tissue swelling and structural lesions with high sensitivity.^31,32^ The use of power doppler helps to assess the vascularity if the synovium indicating an active inflammatory disease.^33^ However, it cannot detect marrow edema^12^ and thus predict erosions like MRI.^34^ EULAR now has recommended USG with power doppler as a sensitive aid to clinical examination in identification of early inflammatory arthritis.^3,25^ High frequency USG with power doppler has been started in Nepal since last six months and has dramatically changed the number of patients diagnosed as undifferentiated arthritis to seronegative inflammatory arthritis requiring methotrexate.

## THERAPEUTIC APPROACH FOR EARLY UNDIFFERENTIATED ARTHRITIS

In the past, early arthritis was treated with non-steroidal anti-inflammatory drugs (NSAIDs) or steroids on as required basis till the patient exhibited clear features of chronicity which then was considered the indication for disease modifying drugs based on risk benefit analysis.^19^ With the current day evidence of damage resulting from delayed treatment, it is unjustifiable to wait for initiation of DMARDs till the evidence of chronicity or erosions become evident.^35–38^ The management goal of current day practice has to be the prevention of such features of damage and chronicity and achieving drug free remission in feasible cases.^3,39^ Once a patient is labelled as undifferentiated arthritis, i.e. after exclusion of other specific forms of arthritis, especially rheumatoid arthritis, assessment should be done for the presence of risk factors for persistence like presence of anti-CCP antibodies, high titer rheumatoid factor, high disease activity, high inflammatory markers or imaging evidence of marrow edema, osteitis, synovial hypertrophy with higher grades of power doppler uptake. ^4^

Evidence has accumulated over time regarding the efficacy of DMARDs in early arthritis even in the absence of classification to any definite form.^3,39,40^ A placebo-controlled trial of methotrexate in early arthritis involving 55 patients in each group used 15mg methotrexate versus placebo and the methotrexate dose was escalated every 3 months depending on DAS score. At the end of 12 months, all patients in placebo group fulfilled the ACR 1987 criteria for rheumatoid arthritis whereas only about half the patients in methotrexate group did. Patients on methotrexate also showed delayed radiographic progression, thus showing the role of methotrexate in treatment of early inflammatory arthritis.^19^ Nell et al compared treatment outcomes with very early arthritis group of less than 3 months duration with those with early arthritis of less than a year with effective doses of methotrexate or sulfasalazine: each group consisting of 20 patients. As early as 3 months of treatment, an improved outcome was evident in the treatment group and the response and radiographic outcomes were maintained at the end of 36 months. ^41^

**Figure 1.**
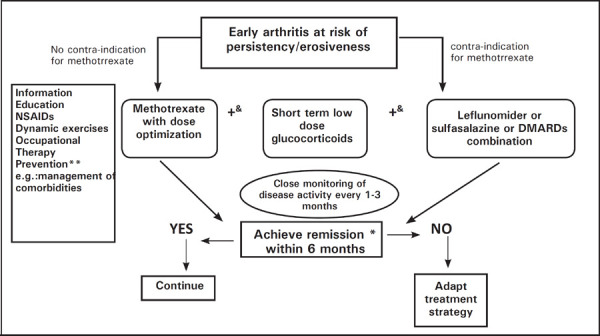
EULAR 2016 recommendation on treatment of early arthritis.^3^

In addition to these small studies, not much data is available on optimal choice of therapeutic agent for cases of EUA. In the presence of one or more high risk features, patients should be started on DMARDs: preferably methotrexate unless contraindicated (like deranged liver function or planning for conception).^3,42–44^ Sulfasalazine or leflunomide can be considered as first line drugs in cases where methotrexate is contraindicated. The clinical efficacy of LEF, and to a lesser extent SSZ, is similar to MTX in established and recent RA.^28^ There is now a definite evidence in favor of glucocorticoid use in low or moderate doses when used in early phase of disease.^45–47^ Steroid use should be tapered once the disease activity is controlled and should be temporary, limited to less than 6months to limit the possible adverse effects of GCs.^3,48^ Patients should be followed every 1 to 3months depending on the disease activity: patients with higher activity being monitored more closely.^28^ If the patient does not achieve significant improvement or remission in 6 months, then the treatment has to be upgraded with combination DMARDs. Either methotrexate with sulfasalazine and hydroxychloroquine or methotrexate with leflunomide can be used depending on patient tolerance, affordability and side effect profiles,^49–51^ though some authors think that the later combination has more toxicity profile.^52–54^ Steroids and NSAIDs can be used for short term in between as bridge therapy during flares and during addition of DMARDs.^3^ Few important studies highlighting the importance of early DMARD treatment along with their outcomes are summarized (Table 1). A therapeutic flow chart from EULAR 2016 updates on recommendation on treatment of early arthritis is shown (Figure 1).^3^ This approach highlights the importance of starting DMARDs early in the course of inflammatory arthritis even if not classified into any specific disease.

Non-pharmacological measureslike patient education on disease outcomes, importance of persistence of treatment,^55^ dental hygiene,^56^ smoking cessation,^57^ weight control^58^ and life style modifications also need to be implemented.

**Table 1:** Summary of treatment outcomes of early inflammatory arthritis.

Study	Population	Characteristics		Treatment	Outcome	Remarks
Lukas C et al ^37^	ESPOIR cohort	- Inflammatory arthritis -6 wks to 6 mths ->2 joints -DMARD or steroid naive	RF-44.5% ACPA- 41 % ACR criteria- 79.4%	MTX- 58% SSZ-13% LEF- 6% Combination- 5.5% HCQ monotherapy not included in study	- 72% no radiographic progression over 1 year - 8% - severe progression (>5 units) - Change in the erosion score at 1 year in 27.1 %	early initiation of DMARD therapy reduces 12 month radiographic progression -significant correlation between
Soderlin et al ^38^	BARFOT study	-> 1 8 yrs age -< 1 yr duration -fulfilling ACR criteria	RF-61 % ACPA-59%	MTX- 47% Any DMARD- 96% GC- 33% Biological- 4%	- disease duration an independent predictor of poor EULAR response at the 1 2 months -patients with a shorter disease duration received less DMARD at 1 2 months	improvement in DAS28, its individual components, and HAQ and disease duration, -patients with shorter disease duration improving most
Nell et al ^41^	VERA-20 pts LERA-20 pts	VERAOmonths LERAOmonth to 1year		MTX> 1 5 mg/wk or SSZ 3g/day	- at 3 months: 40% DAS28 reduction in VERA group 12% DAS28 reduction in LERA group - at 1 yr, DAS28 reached low disease activity among VERA, where it stabilized for the subsequent 2 yr. - Such low mean disease activity was never achieved in the LERA group - a remission-like state (DAS28 <2.6) was seen in 50% of VERA and 1 5% of LERA patients	highly successful treatment of RA in the first year and especially within the first 3 months of therapy
Verschueren^45^	CareRA study	-RA patients fulfilling ACR 1987 -disease duration <1 year -DMARD and Steroid naive		- COBRA Classic: 1 5 mg MTX weekly, 2 g SSZ daily and a weekly step-down GCs COBRA Slim: 15 mg MTX weekly with a weekly step-down GCs COBRA Avant-Garde: 15 mg MTX weekly, 10 mg leflunomide daily & weekly stepdown GCs	Remission at week 16: 70.4% COBRA Classic 73.5% COBRA Slim 68.1 % COBRA Avant-Garde At week 16, a good EULAR response: 79.6% Classic patients 79.6% COBRA Slim 76.6% COBRA Avant-Garde	Classical MTX therapy with bridging GCs at a lower dose than in the original COBRA study as a highly effective and safe remission induction therapy in more than 70% of high-risk patients with early RA

Monitoring of the disease activity in cases of EUA again presents a gray zone in our knowledge. No activity scores or composite measures have been validated for use in cases of undifferentiated arthritis.^28^ Also, there are no defined criteria used to define remission. Terminologies like “no active synovitis” have been used but is not categorical. EULAR recommends the use of composite measures for assessing disease activity and remission.^3,28,59^ Other authors agree using DAS28 score and ACR remission criteria used for monitoring rheumatoid arthritis because most of the cases of EUA are shown to evolve into rheumatoid arthritis which seems to be a logical approach with current day evidence.^60^

## OUTCOMES OF EARLY UNDIFFERENTIATED ARTHRITIS

The outcomes of EUA varies depending on the time zone of the study conducted. Earlier studies in the 1990s and early 2000s recruited patients with duration of less than 2 years as “early” and their treatment strategies were usually mild with pyramid approach except in studies on early use of DMARDs. As the diagnostic armamentarium and therapeutic strategy changed after late 2000s, the outcomes were different as expected. A study of Norfolk registry comparing outcomes of patients enrolled at a difference of 10years showed improvement in swollen joints in the later cohort with no significant difference in tender joint, mortality and functional disability. ^61^ However, even the later cohort was enrolled between 2000 and 2004 when the newer diagnostic modalities were not available and early DMARDs were not a norm. Outcomes of later cohort after 2010 is expected to give different results.

In terms of revision of diagnosis on follow-up, a recent two year outcome study showed that the diagnosis remained as UA in 41% patients and changed to RA in 24% of patients and after two years, arthritis resolved in 59% of EUA patients. ^62^ An unpublished data from Nepal shows that the diagnosis remained as UA in 57% of patients and changed to RA in 34% at 6 months follow-up. Drug free remission at six months were seen in only 4 out of 443 patients completing 6 months follow-up. However, authors communicate that introduction of USG is increasing the percentage of patients being diagnosed as RA. It can be assumed that with new diagnostic modalities, fewer patients are labelled as undifferentiated at the outset currently and of them many are likely to either persist as UA or evolve into RA.

## CONCLUSIONS

The diagnosis of UA is considered when a patient with at least one tender or swollen joint does not fulfil the classification criteria for any other inflammatory rheumatic disease. The chances of persistence can be predicted by the severity of disease at onset, presence of auto-antibodies and suggestive findings on sensitive imaging modalities. Treatment with DMARDs, preferably methotrexate, has to be started as soon as the prognosis is determined with judicious use of steroids or NSAIDs on short term basis. The outcome of EUA depends on the risk factors and therapeutic strategy used and is expected to improve in recent years with easy availability of diagnostic methods in Nepal.

## Conflict of Interest

**None.**
